# Co-administration of extracellular matrix-based biomaterials with neural stem cell transplantation for treatment of central nervous system injury

**DOI:** 10.3389/fnins.2023.1177040

**Published:** 2023-05-15

**Authors:** Eshan B. Damle, Vivianne E. Morrison, Jozef Cioma, Milla Volic, Gregory J. Bix

**Affiliations:** ^1^Clinical Neuroscience Research Center, Tulane University School of Medicine, New Orleans, LA, United States; ^2^Tulane Brain Institute, Tulane University, New Orleans, LA, United States; ^3^Department of Cell and Molecular Biology, Tulane University, New Orleans, LA, United States; ^4^Faculty of Biology, Medicine, and Health, School of Biological Sciences, University of Manchester, Manchester, United Kingdom

**Keywords:** extracellular matrix, neural stem cells, neuroregeneration, biomaterials, CNS development

## Abstract

Injuries and disorders of the central nervous system (CNS) present a particularly difficult challenge for modern medicine to address, given the complex nature of the tissues, obstacles in researching and implementing therapies, and barriers to translating efficacious treatments into human patients. Recent advancements in neural stem cell (NSC) transplantation, endogenous neurogenesis, and *in vivo* reprogramming of non-neural cells into the neuronal lineage represent multiple approaches to resolving CNS injury. However, we propose that one practice that must be incorporated universally in neuroregeneration studies is the use of extracellular matrix (ECM)-mimicking biomaterials to supply the architectural support and cellular microenvironment necessary for partial or complete restoration of function. Through consideration of developmental processes including neurogenesis, cellular migration, and establishment of functional connectivity, as well as evaluation of process-specific interactions between cells and ECM components, insights can be gained to harness and modulate native and induced neurobiological processes to promote CNS tissue repair. Further, evaluation of the current landscape of regenerative medicine and tissue engineering techniques external to the neurosciences provides key perspectives into the role of the ECM in the use of stem cell-based therapies, and the potential directions future neuroregenerative approaches may take. If the most successful of these approaches achieve wide-spread adoption, innovative paired NSC-ECM strategies for neuroregeneration may become prominent in the near future, and with the rapid advances these techniques are poised to herald, a new era of treatment for CNS injury may dawn.

## 1. Introduction

The adult central nervous system (CNS) has limited regenerative potential and cannot effectively mitigate the progression of neurodegenerative diseases. Neuroregenerative strategies based on neural stem cells (NSCs) have been developed to address these shortcomings (Ottoboni et al., 2020), but these tools fail to provide robust, lasting improvements, likely because the mature CNS is not sufficiently supportive of NSCs outside of very specific neurogenic niches (Ma et al., 2009), which provide the appropriate signaling microenvironment and extracellular architecture that NSCs require to proliferate and functionally integrate. However, the outcomes of NSC-based approaches can be improved by the addition of biomaterials that align extracellular environment properties with pro-regenerative processes (e.g., proliferation, survival, migration, connectivity, etc.). For example, conductive polymers paired with electrical stimulation, co-delivered with NSCs, increase functional recovery in a rodent model of ischemic stroke (Oh et al., 2022). To implement this strategy with greater control, we must determine the most effective combinations of NSC-based methods and biomaterials that mimic or change the extracellular environment. The extracellular environment is defined by its extracellular matrix (ECM), a collection of proteins (e.g., collagens, laminins, glycoproteins, proteoglycans, etc.) that define a tissue’s physical properties and signaling capabilities, which can be used to deliver neuroregenerative signals where, when, and how they are needed (Burnside and Bradbury, 2014). To turn this idea into a reality, a deeper understanding of how differential ECM-derived signaling could influence neuroregenerative processes is needed. This approach to tissue repair is also being explored in disciplines outside the neurosciences (i.e., cardiology, orthopedics, bioengineering, etc.), providing a blueprint that may also be applicable to the brain. Given that many neuroregenerative processes have their origins in developmental processes and that ECM proteins and their signaling roles in these processes have been studied extensively, innovative paired NSC-ECM strategies for neuroregeneration may become prominent in the near future.

To spotlight this topic and its relevance to pre-clinical and clinical research on neuroregeneration, we review the current NSC-based strategies and the developmental processes that may guide further investigation of paired NSC-ECM methods. Using β1 integrin as a focal point, we then illustrate why the composition of ECM-like biomaterials requires deep consideration and highlight ECM molecules with neuroregeneration-enhancing potential. Finally, we discuss methods that dramatically improve stem cell-based therapies in pre-clinical animal models, which are used in disciplines outside of the neurosciences but bear a potential relevance to the CNS.

## 2. Interactions between endogenous ECM components and versatile NSC receptors in the immature brain provide targets for ECM-based tool development

### 2.1. Signaling properties of the ECM

ECM-derived signals are often transduced by integrins, a versatile class of heterodimeric proteins, consisting of α and β chains, and flow cytometry has identified the presence of α3, 6, and 7, and β1 and 4 on NSCs (Flanagan et al., 2006). β1-containing integrins constitute the largest integrin subfamily and play a role in NSC proliferation, neuronal migration, and connectivity (Campos et al., 2004, 2006; Porcheri et al., 2014). The versatility of β1 integrin, its ability to integrate diverse environmental cues to guide cell function, and its well-established relationship with the ECM all mean that it is an ideal target for paired NSC-ECM strategies and can demonstrate the widespread impact that an ECM-based approach can have on repairing the injured or diseased brain.

The postnatal subventricular zone (SVZ) contains a specialized ECM structure called a fractone (Mercier, 2016). Fractones are morphologically-complex, extending tendrils of ECM between the ventricle surface—where they are exposed to diverse cerebrospinal fluid (CSF)-borne signaling molecules, such as growth factors—to neural rosettes, in the heart of which are NSCs (Mercier, 2016; Kerever and Arikawa-Hirasawa, 2021). The fractone structure likely serves as a relay station, providing NSCs with the necessary cues for proliferation, the retention of stemness, and survival (Kerever and Arikawa-Hirasawa, 2021). Fractones are composed of proteins found in the basement membrane (BM) that surrounds vasculature, including collagen IV and diverse laminins (Mercier, 2016; Kerever and Arikawa-Hirasawa, 2021), though the SVZ is distinguished from traditional BM by unique heparan sulfate and chondroitin sulfate chains (Kerever et al., 2014) and the heparan sulfate proteoglycans (HSPGs) perlecan and agrin, which are involved in regulating fractone composition and thus, NSC function. For example, the heparan sulfate chains of perlecan are responsible, via their ability to capture fibroblast growth factor 2 (FGF2), for NSC proliferation (Kerever et al., 2014).

Perlecan, as well as its cleavage products, and laminins bind to β1 subunit-containing integrin receptors (Bix et al., 2004), which are highly expressed in the SVZ, especially close to the ventricular surface (Campos et al., 2004). Beyond perlecan and laminins, β1 integrin binds to chondroitin sulfate proteoglycans (CSPGs) called tenascins. Tenascin-C (Tnc) and Tenascin-R (Tnr) are heavily studied in the context of brain development as well as brain pathology, the former being highly expressed in the developing brain and downregulated as the brain matures (Roll and Faissner, 2019). Tnr levels increase as development proceeds and become a central component of perineuronal nets (PNNs), a specialized ECM mesh that forms around neuronal cell bodies (Kazanis et al., 2007; Fawcett et al., 2019).

Tnc is an established ligand for several β1 integrin-containing receptors that are relevant to the neurogenic niche, such as α7β1 (Joester and Faissner, 2001), and the loss of Tnc-dependent β1 signaling leads to NSC processes retraction and reduced viability (Faissner and Reinhard, 2015). Tnc is upregulated following injury, which could be helpful for neural repair (Okada and Suzuki, 2020), but it may not be at sufficiently high levels or at the requisite location to support the proliferation and survival of NSCs. This could be addressed via the implantation of biomaterials that mimic the SVZ ECM and contain higher levels of Tnc than those usually found in non-niche brain regions.

On the other hand, β1 integrin signaling downstream of Tnr activation suppresses proliferation (Liao et al., 2008), which could impede endogenous neurogenesis or the expansion of transplanted NSCs, but if deployed correctly through an ECM-like biomaterial, it could help maintain a pool of NSCs over time, prolonging the neuroregenerative capacity and protecting the NSC pool from exhaustion (Dray et al., 2021). Thus, the introduction of Tnc and Tnr in a manner that is spatiotemporally consistent with how these factors function in the normal brain could modulate β1 integrin signaling on NSCs, improving the expansion of the NSC pool and limiting the risk of NSC exhaustion, which could blunt the positive impacts of endogenous NSC-based strategies.

β1 integrin also plays a role in neuronal migration in the developing brain and again, following injury, its influence could be fine-tuned through the introduction of specific ECM proteins. Reelin, a glycoprotein secreted by Cajal–Retzius cells in the growing cortical plate, is a mediator of cortical lamination and a ligand for α3β1 integrin. Just as tenascins differentially affect NSC proliferation through β1 integrin, reelin signaling through β1 can have several outcomes, though the precise mechanisms of this are still unclear. On one hand, reelin permits migration of newborn neurons past their immediate predecessors, thus contributing to the “inside-out” pattern of cortical growth (D’Arcangelo and Curran, 1998). On the other, reelin can also inhibit neuronal migration (Dulabon et al., 2000). Furthermore, proteolytic processing of reelin yields fragments that may also play distinct roles in regulating neuronal migration (Jossin, 2020; Hattori and Kohno, 2021). Overall, it appears that reelin, through its binding to β1 integrin-containing receptors, plays a nuanced role in determining how immature neurons move through the increasingly complex cortical plate. The modulation of reelin availability, location, and proteolytic processing could be beneficial in the context of injury and degeneration to improve NSC and neuroblast homing to sites of injury.

The interaction of integrins with components of the ECM at the site of injury likely will regulate the formation and fine-tuning of synapses and circuitry, as has been observed in the healthy brain (Lilja and Ivaska, 2018). β1 integrins are enriched at synapses and pair with various α chains to regulate plasticity (Park and Goda, 2016), as evidenced by the fact that loss of β1-dependent signaling impedes synapse formation and ultimately reduces the number of spines in hippocampal neurons (Nikonenko et al., 2003; Webb et al., 2007). Beyond regulating synapse formation and remodeling, β1 integrin is also involved in the composition of neurotransmitter receptors found in the post-synaptic compartment, and it was shown that this phenomenon occurs as a function of an ECM-targeting proteinase, matrix metalloproteinase 9 (MMP9) (Michaluk et al., 2009), raising the possibility that proteolytic processing of the ECM, and thus alterations in the ECM make-up of injury sites and in the degenerating brain, could facilitate synaptogenesis and synaptic function, thus improving integration of new neurons.

### 2.2. Functionality of ECM biomaterials in supporting the neurogenic niche

These processes are complex, and as we discover more about them in the developing brain, it becomes increasingly clear that the NSC-based methods alone cannot achieve the required outcomes. Instead, it is necessary to pair these NSC-based strategies with permissive signaling environments, which could be created via the carefully timed introduction of thoughtfully curated cocktails or sequences of ECM proteins or ECM-modifying proteins to recapitulate native ECM settings at distinct stages of development. Further experimental work into the biostability of introduced ECM components in their new environment is needed to determine precisely how these biomaterials are incorporated in the target tissues as well as whether they persist for the proper duration to allow for cell proliferation, migration, and integration. While ECM degradation is a crucial part of the remodeling process, excessively rapid degradation of injected ECM scaffolding may override the primary utility of introducing exogenous ECM materials—to generate the temporary microenvironment and tissue architecture needed to recreate a pro-neurogenic tissue setting (Tukmachev et al., 2016).

As the ECM and its components provide such a wide range of functions, the potential to induce targeted changes in specific cells based on cell type can be accomplished by selecting the appropriate biomaterial. Hydrogels, meshes, cell sheets, and surface coating are among the biomaterials that have been developed to promote tissue repair throughout the body in animal models and have shown promise in clinical trials (Xing et al., 2020). Engineered cell matrices are being used to administer ECM components such as Tnr, utilizing methods such as the administration of mesenchymal stromal cells via spheroids (Gonzalez-Fernandez et al., 2022). Spheroids can be targeted to a specific site, creating the potential for localized and rapid therapeutic benefits, although the clinical applications are yet to be seen, as human trials have proved difficult (Murphy et al., 2014).

Hyaluronic acid (HA), a critical and ubiquitously expressed ECM glycosaminoglycan, is one of the most frequently used polymers comprising hydrogels (Dalton and Mey, 2009). In order to assess their reparative capability, Hou et al. (2005) implanted crosslinked HA hydrogels, modified with laminin, into mechanically induced cortical lesions in rats. Following 6 and 12 weeks of implantation, brain sections showed that the HA hydrogels shared rheological and mechanistic properties with the native brain tissue. These biomaterials were shown to attenuate inflammation and inhibit glial scar formation while forming a scaffold to support angiogenesis, neurite extension, and engraftment. The anti-inflammatory nature of HA was also shown in a study where HA hydrogels with embedded poly(lactic-co-glycolic acid) microspheres were functionalized with potent angiogenic factors– vascular endothelial growth factor (VEGF) and angiopoietin-1 (Ang1)– and implanted into the ischemic cavities of stroked mice (Ju et al., 2014). Again, the administration of ECM-based biomaterials reduced inflammation and astrogliosis, and clear behavioral improvement was seen in the mice. A favorable niche microenvironment was also established in the ischemic region and angiogenesis was observed, following the release of VEGF and Ang1. Combining NSC transplant strategies with functionalized injectable HA hydrogels could provide the necessary scaffolds to aid the engraftment of stem cells. Prestwich and Healy (2015) emphasize the translational requirement for ECM technologies such as hydrogels and the need to deliver simplicity. Their HA-only hydrogels contain one or two biomolecules and “living” chemistries that allow crosslinking. These types of hydrogels have proven successful in aiding stem cell self-renewal, wound repair, and mitigating post-surgery adhesions.

Due to the extensive involvement of the ECM in various biochemical and signaling processes, the developmental or repair state of certain components must be considered when attempting to utilize biomaterials for therapeutic purposes (Tonti et al., 2021). For example, Tnr, which exists as large polymers near nodes of Ranvier and contributes to the formation of PNNs by organizing GAG chains and binding to CSPGs, is known to be inhibited by the presence of tumor necrosis factor-alpha (TNF-α) and upregulated by platelet-derived growth factor (PDGF) (Anlar and Gunel-Ozcan, 2012). Recent findings suggest that Tnr is involved in the growth of axonal tracts in humans and the production of GABAergic neurons through cytokine and growth factor release, which has been shown to induce plasticity. It is believed that individuals with Tnr deficiencies could lead normal lives, however, these deficiencies can be region specific. For example, it is believed that a Tnr deficiency in the hippocampus can contribute to the pathogenesis of certain epilepsies (Anlar and Gunel-Ozcan, 2012).

The macrostructure of ECM biomaterials can also be adapted for certain applications, with ECM being available in a powdered form that can be solubilized into liquid suspensions or gels (Edgar et al., 2018). While decellularized tissue preserves the vascular landscape and ECM composition, it can be impractical because it does not easily conform to new shapes. This issue can be bypassed using ECM powder-derived constructs, which can seamlessly conform to and fill any cerebral cavity, no matter the geometry. ECM concentration can also be modified through temperature changes, which could be an attractive property in terms of clinical application. Massensini et al. (2015) injected solubilized ECM into rodent stroke cavities, which permeated throughout the peri-infarcted regions and formed a hydrogel at body temperature. This model of ECM delivery allows biomaterials to efficiently conform to the injured tissue in their liquid state and remain structurally sound within the cavity following gelation. The efforts geared toward the technical challenges of biomaterial delivery should be equal to those seen in the research and design of novel ECM-based tools.

## 3. NSC-based approaches are promising, but significant challenges remain

### 3.1. NSC transplantation

NSC transplantation presents distinct advantages but also unique challenges. Culturing NSCs prior to transplantation allows their pre-selection, monitoring, and modification in ways that will support their function *in vivo* but also that of pre-existing host cells. For example, the “secretome” of transplanted NSCs reduces cell death of endogenous neurons (Tang et al., 2017). Despite its advantages, NSC transplantation requires a substantial number of NSCs, presenting a challenge when sourcing these cells. Thus, it would be beneficial to introduce NSCs into environments that support *in situ* proliferation to expand the NSC pool *in vivo* rather than *in vitro* (Tang et al., 2017). Additionally, immunorejection and poor long-term functional integration remain substantial barriers (Li and Chen, 2016). NSCs for transplantation can be harvested from primary tissue samples or generated by the differentiation of pluripotent stem cells (PSCs) or transdifferentiation of non-neuronal cells (Tang et al., 2017).

Primary NSCs are isolated from the subependymal zone (SEZ) of the forebrain [sometimes called the subventricular zone (SVZ)] (Morshead et al., 1994), the olfactory bulb (OB) (Pagano et al., 2000), and periventricular regions of the spinal cord (Mothe and Tator, 2015). The cells are then cultured in treated, conditioned, or selective culture medium and subcultured to expand the NSC pool (Ferrari et al., 2010). PSC-to-NSC differentiation falls under one of two categories: embryonic stem cell (ESC)- or induced pluripotent stem cell (iPSC)-based. To an even greater degree than for primary NSCs, these approaches allow significant control over cell characteristics and function (De Gioia et al., 2020), minimizing the risk posed by incomplete differentiation, such as teratoma formation (Pruszak et al., 2007). However, the manufacturing process for iPSCs is complex, and standardized methods of evaluating the transformation from iPSC to NSC are still being established, presenting a barrier to reliable and replicable experimentation (Sullivan et al., 2018). Finally, ethical considerations relating to human ESCs may prevent or restrict their use as NSC precursors.

Transdifferentiation-based techniques generate NSCs from the reprogramming of mature somatic cells *in vitro*. Growth factor- and transcription factor-induced transdifferentiation incorporates principles of molecular signaling with carefully controlled cellular environments to alter cell lineage and conversion to stem cell fates (Thier et al., 2012; Tang et al., 2017; Mollinari et al., 2018). Finally, chemical compound-induced transdifferentiation employs chemical treatments to convert cellular lineage via largely unknown mechanisms (Tang et al., 2017). Each method has benefits and drawbacks. For instance, transcription factor-induced transdifferentiation relies on exogenous, virus-mediated gene expression, a limitation that chemical-induced transdifferentiation does not pose (Pereira et al., 2019). Overall, transdifferentiation presents a powerful tool for studying the therapeutic potential of NSCs, however, the technique is still evolving, making its use less appealing in patients at this time.

With the survival or engraftment rates of stem cells being <5% *in vivo*, successful NSC transplantation and tissue regeneration cannot occur without the support of biomaterial platforms (Zhang et al., 2019). Improving the proliferation, differentiation, and retention of transplanted stem cells *in vivo* is crucial for their integration, and reducing their dispersion away from the target areas is necessary to achieve maximal therapeutic effect. Poor engraftment can be attributed to the general lack of cell–cell and cell-ECM crosstalk, as well as the cell death signals released by reactive glial cells and peripheral leukocytes (González-Nieto et al., 2018). These issues have been addressed through the pre-conditioning and “functionalized ECM” biomaterial-assisted transplantation of stem cells, with the sequestration of growth-factors and bioactive cues having unparalleled advantages for directing stem cell fate (Hettiaratchi et al., 2016).

Many morphogens, including VEGF, Insulin-like Growth Factors (IGFs), Fibroblast Growth Factors (FGFs), Bone Morphogenic Proteins (BMPs), Epidermal Growth Factor (EGF), and more, have been found to act as proliferation signals for NSC maintenance within the post-injury niche brain microenvironment. Lu et al. (2003) also showed that various neurotrophic factors, such as Brain-Derived Neurotrophic Factor (BDNF), Nerve Growth Factor (NGF), and Glial cell line-Derived Neurotrophic Factor (GDNF), are secreted by NSCs in order to promote neurogenesis following injury. GDNF and BDNF were incorporated into electrospun polycaprolactone-based scaffolds, which were co-administered with transplanted NSCs. These functionalized scaffolds were shown to have enhanced NSC survival and engraftment both *in vitro* and *in vivo* (Bruggeman et al., 2017; Nisbet et al., 2018). The release of GDNF increased NSC proliferation and differentiation, while also attenuating the astrocytic inflammatory response. Furthermore, following stroke or a traumatic brain injury, the harsh microenvironment can be re-engineered via biomaterial-mediated cytokine and chemokine release. As transplanted cells lack immunomodulatory function, Transforming Growth Factor- β (TGF- β ) and other cytokines can be added to ECM powders or tethered to microporous scaffolds to reduce the pro-inflammatory response once they are administered. Promoting the anti-inflammatory response can increase the survival, retention, and engraftment rates of transplanted stem cells, and reduce their chances of immunorejection (Liu et al., 2016; Edgar et al., 2018).

### 3.2. Endogenous neurogenesis

Both the hippocampus and the SVZ produce NSCs in the adult brain, though less so than in the developing brain (Patel and Sun, 2016). Administration of growth factors, neurotrophic factors, and hormones; pharmacologic induction of epigenetic modifications; as well as modulation of other signaling processes such as the Notch signaling pathway have been shown to influence adult endogenous neurogenesis (Imayoshi et al., 2010; Xiong et al., 2011; Shohayeb et al., 2018; Baumann et al., 2019). Alternative approaches using small molecule-mimics of endogenous factors also exist (Kazim and Iqbal, 2016) and may translate more effectively to pharmacologic interventions (Patel and Sun, 2016). Interestingly, even physical and electrical stimulation have been shown to promote endogenous neuroregeneration, examples of which range from physical exercise to deep brain stimulation to targeted laser therapies (Piao et al., 2013; Xuan et al., 2015; Tsai et al., 2019). Finally, others suggest that administration of biomaterial scaffolds, along with relevant growth factors, may be sufficient to spur endogenous neurogenesis and promote brain tissue recovery (Collins et al., 2022).

Though the potential for manipulating endogenous neurogenic processes is promising, some limitations of these methods remain. Namely, the regionally restricted locations of NSCs pose a challenge, as the developing brain, with all its migratory streams and signaling cues, differs substantially from the mature brain (Kaneko et al., 2017). The absence of selection and modification steps that exist for NSC transplantation means there is a greater risk of biological processes unfolding in unforeseen ways, potentially leading to unintended consequences or failure to reach desired outcomes. However, recent investigations into the use of tissue engineering and biomaterial-based strategies to manipulate endogenous neurogenic processes have advanced the ability of researchers to influence relevant molecular and cellular processes. This indicates that these techniques hold great promise for 1 day generating a neurogenic strategy that can be implemented in human patients experiencing CNS injury (Purvis et al., 2020).

Current approaches for stimulating endogenous neurogenesis largely focus on the direct manipulation of NSCs, but it is necessary to consider another aspect of the equation: the architectural support, biosignaling relevance, and provision of an ideal microenvironment that the ECM contributes (Chen et al., 2022). Future approaches could test whether co-administration of ECM-like biomaterials provides a suitable environment for neural tissue repair and neuroregeneration.

### 3.3. *In vivo* reprogramming

*In vivo* reprogramming involves the conversion of endogenous non-neuronal cells into target neuronal cell lineages. This differs from NSC transplantation in that it modifies the organism’s own cells into the desired cell type (Li and Chen, 2016; Fang et al., 2018). Further, specific cell types can be selectively reprogrammed, altering their morphologies and functionalities to adapt to diseased or injured conditions. These techniques aim to harness existing cell populations in affected regions to restore functionality of brain parenchyma (Wei and Shetty, 2021).

A significant advantage of this approach is the avoidance of complications caused by immunorejection and mitigation of challenges with functional integration (Li and Chen, 2016). The granular level of control presented by *in vivo* reprogramming allows for the targeted conversion of specific cell types into neurons using a variety of highly specific chemical and signaling factors (Tai et al., 2020). Further, such pinpoint approaches may allow for control over the cortical layer-specific identities of resultant neurons, which may play a role in eventual functional connectivity and the extent of successful integration into neural circuitry (Mattugini et al., 2019). Additional advantages include the relatively time-efficient nature of the technique, which may be relevant when neuronal remodeling over time is a concern, minimal ethical concerns, and minimal risk of tumorigenesis induced by the technique itself (Sekiryu and Matsuda, 2021). Interestingly, it has even been suggested that the reprogramming process may reverse some aspects of cellular aging (Rohani et al., 2014).

Still, *in vivo* reprogramming presents several challenges and risks. The conversion of a portion of the already-limited number of surviving cells post-injury into neurons may lead to a depletion of the endogenous cell population (Fang et al., 2018). Further, the potential for genetic mutations in reprogrammed cells may increase the risk of tumorigenesis, and this technique may also be less suitable for addressing genetic neurodegenerative diseases. Selection of suitable delivery systems must also be considered, given the trade-off between the ability to transport large inserts with the risk of integration into the host genome and disruption of other genes (Fang et al., 2018). One proposed workaround utilizes small molecules to reprogram cellular fates, which are easily synthesized and manipulated, offer high cell permeability, and often have reversible regulatory effects on protein function. While small molecules already provide significant spatiotemporal control over protein activation and/or inhibition, further calibration of their concentration allows for the regulation of several targets across multiple protein families in order to induce an advantageous phenotype (Xu et al., 2008; Li et al., 2018). This method requires further investigation due to both the specificity and the novelty of such techniques, with many small molecules remaining dependent on exogenous factors in order to induce complete cell fate conversion (Xu et al., 2015). Finally, the relative difficulty of implementing and monitoring the *in vivo* reprogramming process is a worthwhile consideration, as the ability to translate therapeutics, even if successful, into human patients without a means of actively monitoring and controlling the process may pose a problem.

Following any NSC-based transplantation or reprogramming strategy, successful tissue regeneration is dependent upon successful cell engraftment. ECM-based biomaterials could be utilized to enhance the engraftment of reprogrammed cells by providing the necessary neurogenic niche for stem cell adhesion, proliferation, differentiation, and survival. ECM-based biomaterials, such as scaffolds and hydrogels, can mimic the architecture of the ECM, provide physical support to damaged tissues, and promote cell attachment and proliferation. They can also be functionalized to release growth factors, drugs, anti-inflammatory factors, bioactive molecules, and other important proteins sequestered *in situ* (Collins et al., 2022). These cues can be used to help upregulate signaling pathways and vascular regeneration mechanisms, subdue the pro-inflammatory responses that may harm developing cells, and simply modulate the injury microenvironment to strengthen the chances of stem cell retention and engraftment within the reprogrammed region.

In order to induce functional tissue repair following focal ischemic stroke or traumatic brain injury in mice, native cells like astrocytes can be reprogrammed *in vivo* into NSCs or neurons via de- or trans-differentiation, respectively (Ma et al., 2021; Peng et al., 2022). Astrocytes tend to proliferate and populate penumbral tissue encompassing the lesion or infarct site, which makes them the ideal target for such strategies. Unfortunately, astrocytes enter a “reactive” state following injury, eliciting an astrocytic inflammatory response through the release of various nanofilament proteins. This encloses the lesion to form a glial scar and prohibits axon regeneration (Pekny et al., 2014; Liddelow and Barres, 2017). In such a harsh inflammatory microenvironment, the turnover and survival rate of reprogrammed NSCs can be low, as neuronal maturation cannot be supported solely by the surrounding conditions (Hao et al., 2014; Kim et al., 2020). Biomaterial scaffolds, predominantly applied as hydrogels, could be used to replicate the healthier native microenvironment in order to support tissue regeneration and cell engraftment in the reprogrammed region (Wang et al., 2018). Hydrogels can be used to plug lesion site cavities, deliver bioactive molecules, and act as architectural support in damaged tissue that encourages cellular interactions and neural network crosstalk (González-Nieto et al., 2018). Hydrogel porosity, gelation time, and polymer composition can be fine-tuned, allowing the gel and its encapsulated neurotrophic factors to be injected directly into the ischemic core or the surrounding peri-infarction region (Ghuman et al., 2016; Cook et al., 2017). This provides a scaffold in which the reprogrammed penumbral cells can integrate and, depending on the enclosed factors, promote the survival of NSCs and the re-establishment of functional connectivity.

**Figure 1:**
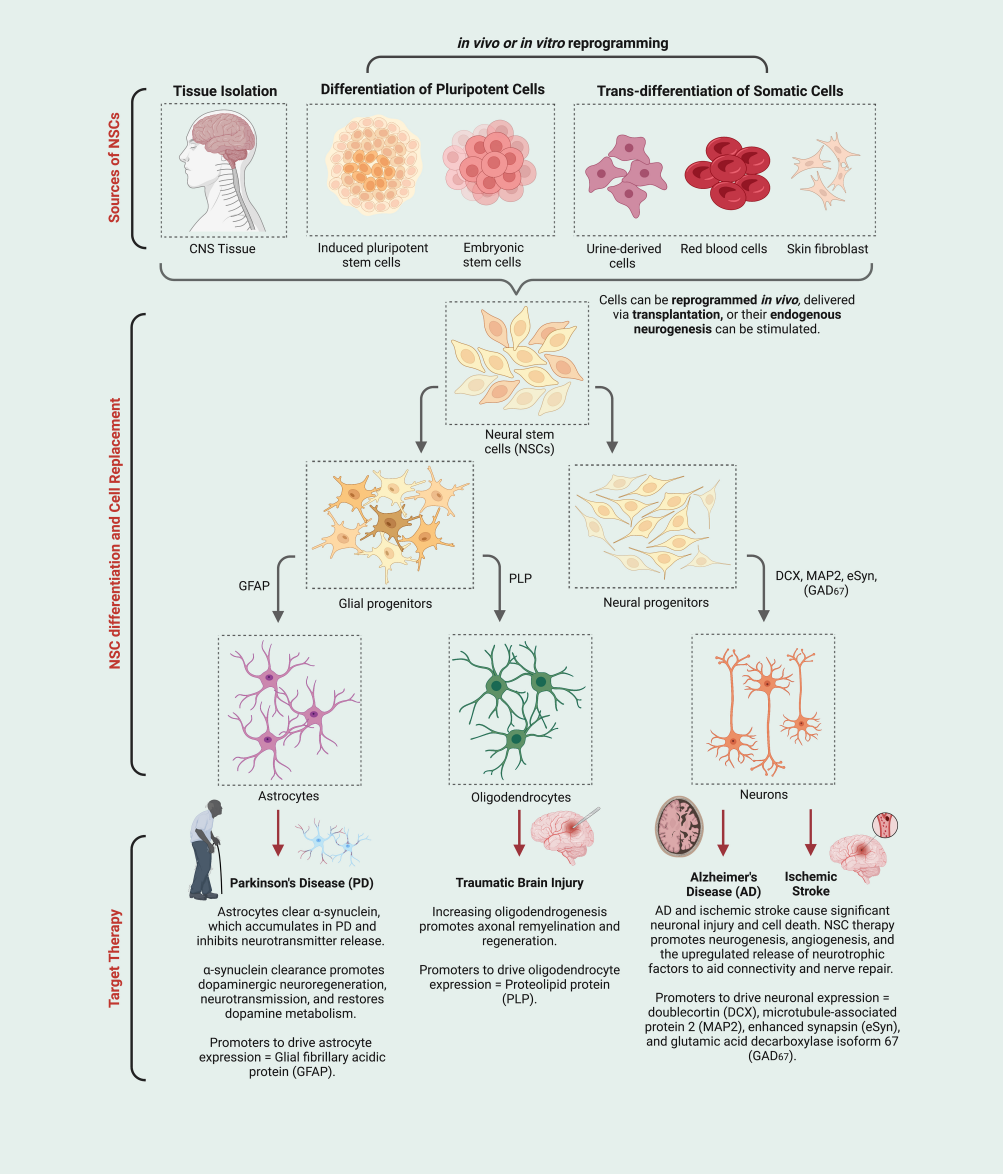
Neural stem cells (NSCs) derived using multiple techniques (e.g., stimulation of endogenous NSCs, induced pluripotent stem cells, etc.) give rise to a variety of neural cell types (i.e., astrocytes, oligodendrocytes, and neurons) that can be deployed in ways that could address specific neurodegenerative conditions or injuries (i.e., new astrocytes may increase clearance of neurotoxic protein aggregates; new oligodendrocytes and remyelination may provide neuroprotection for damaged or stressed neurons; new neurons may replace those that have degenerated and can help re-establish connectivity and tissue repair directly and indirectly.

While there is an abundance of literature on how biomaterials can improve stem cell transplantation and *in vitro* reprogramming, there seems to be a lack of research on the implementation of ECM-based biomaterials to aid the engraftment of cells reprogrammed *in vivo*. The current NSC-based approaches are promising but have some very real drawbacks at multiple steps in each case. However, some of those weaknesses, such as limited ability to control or monitor NSC function *in vivo,* could be addressed by the addition of ECM-based tools. The nature of the ECM in these tools must be considered carefully and in a process-specific way. The developing CNS, therefore, serves as a blueprint for these questions and provides several targets that could easily and rapidly be tested for their potential to enhance current NSC-based techniques (Figure 1).

## 4. Developmental processes may address the weakness of current NSC-based neuroregenerative and neuroprotective approaches

### 4.1. Neurogenesis

NSCs in the SVZ, arising of highly varied origin largely from the ventral brain (Young et al., 2007), contribute to fetal cortical development, producing neuroblasts that become excitatory projection neurons in the cortex (Franco and Müller, 2013). In the postnatal mouse, NSCs contribute largely to olfactory bulb neurogenesis (OBn), by which hundreds of thousands of neuroblasts are generated every day and subsequently migrate to the OB, becoming inhibitory interneurons (Lim and Alvarez-Buylla, 2016). Cells that could be considered “true NSCs” (also called type B cells) insofar as their epigenetic landscape resembles that of stem cells in other tissues, proliferate slowly and, consequently, their proliferative capacity is prolonged, resulting in a pool of NSCs that persist in the adult brain (Lim and Alvarez-Buylla, 2016). In gyrencephalic organisms (e.g., humans, non-human primates, ferrets, etc.), neuroblast numbers are dramatically increased in a region called the outer SVZ (oSVZ), and intermediate progenitor cells (IPCs) in the oSVZ are transcriptomically distinct from those in the inner SVZ (Fietz et al., 2012; Toda et al., 2016). In infant humans, SVZ neurogenesis occurs to a significant extent but largely diminishes in the first few years of life (Sanai et al., 2011) except in disease contexts, where SVZ-derived newborn neurons have been observed in the subcortical and cortical regions (Arsenijevic et al., 2001; Bernier et al., 2002; Kang et al., 2011; Jhaveri et al., 2018; Sorrells et al., 2019), raising the possibility that regenerative potential exists in the human adult SVZ and could be enhanced by ECM-based approaches.

In particular, the pro-neurogenic ECM function of the oSVZ could be recreated via an implantable biomaterial that is capable of the regulated delivery of LGALS3BP, a secreted NSC-derived protein that interacts with the ECM in the oSVZ and plays a critical role in determining the identity and location of SVZ-derived NSC/IPCs, as well as in constructing complex ECM environments, as evidenced by the role of LGALS3BP in cortical folding (Kyrousi et al., 2021). In modulating LGALS3BP abundance via implantable biomaterials, it may be possible to enhance NSC/IPC proliferation, thus addressing the existing challenge of generating sufficient NSC numbers to support regeneration in the injured brain. This would be advantageous for supporting neuroregeneration following trauma, in which large swaths of brain tissue are damaged, placing a higher demand on NSCs to produce neurons. Also, it is conceivable that LGALS3BP could be leveraged to influence cell fate decisions, thus determining what kind of neurons are made available to the regenerating brain, but the application of this idea has not yet been attempted, to our knowledge. It may be invaluable for neurodegenerative diseases that affect a specific region of the brain such as the basal ganglia in Parkinson’s Disease and Huntington’s Disease. There is a great need to determine to what extent leveraging oSVZ properties and functions can improve regeneration and repair, in particular because it is a structure found in humans, not in rodents, and thus may not face the translational barriers faced by neurogenic structures that exist in mice but not humans, such as the rostral migratory stream (RMS) that feeds new neurons to the OB. The oSVZ may prove critical for generating enough NSCs and neurons to meet the needs of the injured or degenerating brain.

Beyond sufficient NSC proliferation and neuron production, the survival of newborn cells is key. Even in the healthy brain, many neuroblasts exist for only a short period of time before they undergo apoptosis and are cleared by CNS-resident macrophage-lineage cells (microglia), all within a few days of being born (Sierra et al., 2010). Neuroblast turnover is a tightly regulated process, and disruptions in the process negatively impact neurogenesis (Diaz-Aparicio et al., 2020), though many aspects of the process and the mechanisms that control it are still unclear. As more is discovered about how microglia, which express ECM-modifying proteases (Crapser et al., 2021), influence neuroblast survival, it may be useful to integrate that into ECM-based tools, as well.

Thus, the net success of neurogenesis depends on NSC/IPC proliferation as well as neuroblast survival. Additionally, given that NSCs are multipotent and can also give rise to glia, neurogenesis also relies on the orchestration of signals that are pro-neurogenic rather than pro-gliogenic. Indeed, alterations in NSC function can shunt IPCs/newborn cells toward glial fates at the expense of the neuronal lineage (Sugimori et al., 2007). Therefore, the proliferative capacity, survival, and fate of NSCs and their progeny, whether endogenously generated or transplanted, are three aspects that must be considered when developing ECM-based strategies to promote neurogenesis.

### 4.2. Neuronal migration

If NSC-based therapies are to be successful, then neuroblasts, either endogenously generated or transplanted, must quickly reach injury sites. Thus, any NSC- and ECM-based strategy must take into account the elements that are necessary to divert NSCs and neuroblasts away from physiological migration routes (e.g., of OBn) and toward injury sites. Consideration of developmental migratory patterns, like those responsible for corticogenesis and OBn, may prove crucial to developing novel pro-regenerative tools.

The layering pattern of the cortex results from neuroblasts being ushered to the correct location prior to maturation. If neuronal positioning is altered, it severely disrupts cortical organization and brain function (Selemon et al., 1995; Gressens, 2000; Tuoc et al., 2009). Early-born neurons travel a short distance, radially from the SVZ, to populate the growing cortical plate, constituting the deep cortical layers. With each round of neuroblast generation, the cells migrate beyond the deeper layers and then stop, creating the characteristic cortical layers in an “inside-out” fashion (Agirman et al., 2017). Neuroblasts born from the ganglionic eminences, which make up the ventrolateral surface of the ventricle migrate tangentially (i.e., in a coronal plane) into the cortex (Barber and Pierani, 2016). These main thoroughfares of neuronal migration become less prominent as gestation ends and as glial cell types, as well as postnatal OB-bound neuroblasts, appear.

Postnatal neuroblasts bound for the OB in mice perform longitudinal migration, traveling rostrally from the SVZ toward the OB in the RMS (Lim and Alvarez-Buylla, 2016). The RMS, with its size and ability to support massive numbers of migrating cells, may harbor key details about successfully relocating cells from deep brain regions into outer-lying cortical regions, and about how to maintain an immature identity until arrival at the final destination. A successful ECM-based tool, therefore, could greatly improve neuroblast homing to lesion sites by repurposing these known migration routes, though further experimental work needs to be conducted to elucidate the exact composition and administration schedule of biomaterial components to foster targeted localization most successfully (Belvindrah et al., 2007). Thus, the ECM that defines migration routes in the developing brain could be a valuable tool in improving NSC-based interventions.

### 4.3. Connectivity

A successful paired NSC-ECM-based strategy would ensure that new neurons could form functional circuits in and around sites of injury and areas of neurodegeneration. For many neural circuits, crucial connectivity is established via experience-dependent plasticity occurring in a specific developmental window, a “critical period” (Reh et al., 2020). Within the critical period, neurons have an increased sensitivity to changes in the environment, leading to rapid and robust changes in connectivity. In the time before or following a critical period, however, there is limited potential for synaptogenesis and plasticity (i.e., the formation and remodeling of synaptic connections) at the scale that is seen in these developmental phases. This restriction of plasticity is due in part to the formation of PNNs, which are found surrounding parvalbumin (PV)-positive GABAergic neurons (Hübener and Bonhoeffer, 2014). The degradation of the PNN surrounding PV neurons has been shown to correlate with similar neural plasticity levels as those observed in critical periods. Conversely, increasing levels of myelination and the development of perinodal ECM (i.e., surrounding the nodes of Ranvier that are responsible for saltatory conduction) limits plasticity (Fawcett et al., 2019). Thus, the manipulation of peri-lesional ECM to recreate a “critical period-like” environment may improve the long-term success of NSC-based strategies by increasing the likelihood that newborn neurons become integrated into pre-existing circuitry.

Injuries of the CNS and spinal cord have been targeted therapeutically through the promotion of plasticity. Axonal sprouting to treat spinal injuries has been demonstrated by using the bacterial enzyme chondroitinase ABC to digest PNNs, allowing plasticity to occur on a larger scale (Zhao and Fawcett, 2013). Adult NSCs from the spinal cord have also been shown to integrate and differentiate when complemented by FGF2 after being transplanted to the dentate gyrus (Shihabuddin et al., 2000). The study of ECM material integration in the treatment of injuries has been demonstrated in many different tissue types and extensively discussed, as by Hussey et al. (2018). The extent to which variations in disease, injury, and tissue type affect the connectivity of ECM materials is wide-ranging, and examination of each would move beyond the scope of this review. However, it should be noted that technologies such as hydrogels and porous polymeric networks that lend themselves to stem cultures can potentially target injuries such as traumatic brain injury and stroke by influencing connectivity among other physical properties (Yin and Cao, 2021).

## 5. ECM-based strategies from outside the neurosciences could be leveraged in the brain

ECM-based strategies for tissue repair are not limited to the neurosciences; in fact, the development of such strategies has been the focus of much research within the worlds of tissue engineering, for *de novo* tissue formation, and regenerative medicine, as a means of treating injury to existing tissue (Edgar et al., 2018; Assunção et al., 2020). The deployment of minimally invasive ECM-based injections for injury treatment is of particular relevance to ECM-based neuroregeneration research. In alignment with what is known about current NSC-based strategies, the introduction of non-neuronal tissue-specific progenitor cells without supporting ECM architecture leads to poor survival and engraftment *in vivo* (Prestwich and Healy, 2015). Though different from CNS parenchyma, it is worth examining a few examples of simultaneous NSC and ECM deployment from the current research landscape outside of the neurosciences to assess what strategies could be applicable to CNS regeneration. Examining these successful approaches may yield insights into how specific limitations of existing neuroscience strategies, including but not limited to challenges with vascularization, cell survival and integration, and differentiation, may be overcome.

Several studies have been conducted with the aim of enhancing revascularization of injured tissue outside the CNS. Jeong et al. (2018) injected decellularized ECM (dECM) alongside mesenchymal stem cells (MSCs) to promote angiogenesis in a rat model of ischemia in hindlimbs. Rao et al. (2017) enhanced myoblast retention, enhanced engraftment, and improved perfusion of skeletal muscle progenitor cell (myoblast) transplantation in a rat model of ischemic muscle injury using an altered version of skeletal muscle ECM (SkECM) in a nanofibrous hydrogen form, which was easily injectable but also structurally appropriate. Kim et al. (2022) used human pluripotent stem cells (hPSCs) co-administered with decellularized extracellular matrix (dECM) to improve vascularization and maturation of kidney organoids transplanted into mice. These strategies improved grafting and angiogenic factor expression, increased microvessel formation, and reduced fibrosis, demonstrating the superiority of a paired stem cell-ECM approach over administration of stem cells alone to promote revascularization.

Another challenge in current neuroscience strategies lies in ensuring cell survival post-transplant. Zeng et al. (2022) showed that injected dECM from pig cartilage improved the differentiation of human urine-derived stem cells (USCs) into chondrocytes, supported USC proliferation and survival, enhanced cartilaginous ECM production *in vitro*, and improved cartilage regeneration in rat models of cartilage defect. In addition to promoting angiogenesis, He et al. (2015) promoted proliferation and inhibited apoptosis of human embryonic stem cell-derived endothelial cells (hESC-ECs) administered in a mouse myocardial infarction model using Matrigel, a reconstituted basement membrane that mimics the ECM. Shafiq et al. (2021) discussed the mechanisms by which biomaterial-based systems enhance stem cell proliferation and integration by way of specific bioactive cues, demonstrating the crucial role played by the signaling microenvironment and the potential uses of co-administered matrix components. These studies suggest that co-administration of supportive architecture enhances cell survival while also promoting normal functional and structural development to increase tissue repair.

Given the crucial role played by the cellular microenvironment and local cues in determining the fates of transplanted stem cells, it follows that thoughtful administration of ECM components can allow for the modulation of the differentiation process. Xu and Guan (2016) discussed the targeted utilization of various biomaterial properties to determine stem cell fates and generate cardiomyocytes following ischemic injury. Imamura et al. (2004) determined that 3D biomaterial systems with engineered ECM effectively promoted differentiation and maturation of pluripotent embryonic stem cells into hepatocyte-like cells (HLCs). Leijten et al. (2016), while exploring one-cell versus microaggregate strategies for chondrogenesis, implemented a cell-laden biomaterial-based therapy to promote differentiation of human periosteum-derived stem cells into chondrocytes. In tapping into the signaling pathways that stem cells rely on for determining cell fates, biomaterials may alleviate the difficulties faced in CNS-based therapies, as can be seen by the successful implementation of biomaterial-based strategies in non-CNS studies.

There are a plethora of experiments demonstrating that the co-introduction of ECM biomaterials during stem cell transplantation improves viability and engraftment. The ability for a synthetic ECM (sECM) to be injected may allow for ligand and growth factor presentation, enzymatic degradation, material mechanics, and matrix architecture to be modified to optimize therapeutic benefits. Further, genetically modified stem cells are able to produce beneficial structural and signaling products, and these cells are significantly affected by the presence and composition of the ECM (Pollock and Healy, 2009) and the microenvironment it supplies. A cursory glance at the available work suggests that the closer the various components of the “therapeutic cocktail” or infusion schedule (to be administered in distinct stages as appropriate for specific developmental milestones) are to including the complete make-up of cellular and extra-cellular components of the relevant tissue *in vivo*, the more successful the implementation of these therapies tend to be. As such, it seems plausible that co-administration of ECM materials with NSCs is a minimum, and more research should be done to examine the various combinations of materials to identify the ideal composition of therapeutic components. In implementing such a strategy in a highly individualized manner, we may 1 day exist in a world where neurological injury can be treated, or at least attenuated, by way of merely injecting an infusion schedule of NSCs, ECM components, relevant supportive progenitor cells, and appropriate signaling factors.

## 6. Discussion

The use of NSCs in the treatment of CNS injury holds great promise, but further research is necessary to establish the exact methodologies of implementing NSC-based therapies. One aspect we believe is of critical importance to future work is the implementation of biomaterials to reproduce native ECM structures and re-establish the many crucial functions of the ECM. Such functions include, but are not limited to, signaling for the coordination of differentiation and migration, organization of neuronal connectivity patterns, and promoting the proliferation of supportive cells necessary for effective neuroregeneration. By pairing thoughtful ECM component selection with knowledge of various neuroregenerative processes (i.e., neurogenesis, synaptogenesis, etc.) and, further, regeneration of supporting architecture [i.e., angiogenesis, astrocyte remodeling (Sun and Jakobs, 2012), etc.] following CNS injury, a more modular control over the regenerative process can be established, and all avenues can be explored to give the best chance of restoring normal structure, connectivity, and function.

One aspect of the ECM biomaterials approach that requires further investigation is the functional differences between paired NSC-ECM biomaterials strategies (as is the case with dual NSC transplantation-ECM biomaterial administration) and paired acellular factor-ECM biomaterials approaches (as is the case with endogenous neurogenesis and *in vivo* reprogramming partnered with ECM biomaterial administration). The potential for functional advantages with approaches that allow NSCs to enmesh into the biomaterial framework prior to introduction into target tissues (potentially giving them a “head start”) suggests that these approaches may not all be equal, and thus further study to provide insights into the respective efficacies of these targeted therapies will inform future incorporation into medical treatment strategies.

Further, questions about the practicality of these techniques are of particular note; after all, strategies that prove effective in animal models but are impractical or impermissible in humans are of minimal use. Concerns about how to successfully administer therapies with physical and material properties (i.e., occupying space in regions known to be volume-restricted) merit exploration. Potential approaches could include administration in therapeutic installments over time as opposed to a bolus, and with regional spacing as opposed to a single target location (George et al., 2018), but this only raises more questions. For instance, given the often time-sensitive nature of acute CNS injury, what is the effective window within which treatment must be administered in order to safeguard against long-term deficits? Additionally, what, if any, are the ideal locations for administration within the brain tissue? Further, does the location and nature of injury change the ideal treatment strategy? And finally, how will findings from rodent models differ from treatments likely to be approved for human patients, and what additional considerations does the transition from rodent models to humans necessitate?

In learning from other fields exploring similar implementation of stem cells for regenerative purposes, we can see that the data points to co-introduction of ECM analogs with stem cells to promote engraftment and restoration of function. The limited current research utilizing this approach within nervous tissue concurs, demonstrating higher rates of successful integration and minimization of functional deficit. As such, we believe that the future of research into neuroregeneration as a treatment for CNS injury will involve co-administration of ECM-analogous biomaterials alongside a partner cell-supplying or modulating approach to provide the relevant tissue architecture and cellular microenvironment for the normal proliferation, differentiation, and synapse formation of newly-forming neurons.

## Author contributions

VM and GB developed the topic of the manuscript. ED and VM researched and wrote the initial manuscript. ED, JC, and MV wrote additional sections during revisions. MV generated Figure 1. ED, VM, JC, MV, and GB reviewed and edited the manuscript. All authors contributed to the article and approved the submitted version.

## Conflict of interest

The authors declare that the research was conducted in the absence of any commercial or financial relationships that could be construed as a potential conflict of interest.

## Publisher’s note

All claims expressed in this article are solely those of the authors and do not necessarily represent those of their affiliated organizations, or those of the publisher, the editors and the reviewers. Any product that may be evaluated in this article, or claim that may be made by its manufacturer, is not guaranteed or endorsed by the publisher.
